# STABILITY (Symptomatic Review during Biologic Therapy) of Inflammatory Bowel Disease Patients Receiving Infusion Therapy Improves Clinical Outcomes

**DOI:** 10.3390/pathophysiology31030030

**Published:** 2024-08-12

**Authors:** Kelli Morgan, James Morris, Qiang Cai, Phillip Kilgore, Urska Cvek, Marjan Trutschl, Katelynn T. Lofton, Meher Sindhoora Mavuram, Prerana Ramesh, Nhi Dao, Ahmed Alhaque, Jonathan Steven Alexander

**Affiliations:** 1 Department of Gastroenterology and Hepatology, Louisiana State University Health Shreveport, Shreveport, LA 71103, USA; kelli.morgan@ochsner.org (K.M.); james.morris@lsuhs.edu (J.M.); qiang.cai@lsuhs.edu (Q.C.); meher.mavuram@tddctx.com (M.S.M.); 2 Department of Computer Science, Louisiana State University Health Shreveport, Shreveport, LA 71103, USA; pkilgore@lsus.edu (P.K.); ucvek@lsus.edu (U.C.); mtrutsch@lsus.edu (M.T.); 3 Department of Molecular & Cellular Physiology, Louisiana State University Health Shreveport, Shreveport, LA 71103, USApcr002@lsuhs.edu (P.R.);

**Keywords:** ulcerative colitis, UC, Crohn’s disease, CD

## Abstract

Several studies have correlate improved patient outcomes with increased physician–patient contacts, particularly in chronic diseases. Extending this approach to inflammatory bowel disease (IBD) care presents a promising means of improving outcomes. At LSU Health Shreveport (LSUHS), a new approach called “STABILITY” (Symptomatic Review during Biologic Therapy) was implemented during infusion therapy visits for IBD patients. These brief 15 min physician–patient interviews aimed to discuss the patients’ current IBD-related symptoms and evaluate the need for any changes in their treatment plan. Our goal was to remove a care gap and prevent intensifying symptoms created by missed appointments and loss of contact. To analyze the effectiveness of the STABILITY approach, a retrospective chart review was conducted on 111 IBD patients (18 with ulcerative colitis, 93 with Crohn’s disease) seen at LSUHS between 2011 and 2022. Since March 2019, STABILITY has been mandatory for all infusion therapy visits. The data collected included patients’ demographics, lab levels for biomarkers (fecal calprotectin, C-reactive protein, and erythrocyte sedimentation rates), hospitalizations, medication changes, and diagnosis dates before and after the implementation of STABILITY. Additionally, voluntary, anonymous infusion patient satisfaction surveys post-STABILITY were used to gather patient responses. In males with IBD, disease severity and hospitalizations were reduced significantly (*p* = 0.004 and 0.0234, respectively). In females with IBD, disease severity and hospitalizations were also reduced significantly (*p* = 0.0001 and 0.0072, respectively). In patients with UC and CD, there were significant improvements in disease severity (*p* = 0.043 and *p* = 0.0001, respectively), and CD hospitalizations were also improved (*p* = 0.0013). In males and females with UC, disease severity was marginally and significantly reduced (*p* = 0.0781 and *p* = 0.0379, respectively). In males and females with CD, disease severity was significantly reduced (*p* = 0.0161 and 0.0003, respectively), and CD male and female hospitalizations were also reduced significantly (*p* = 0.0436 and 0.013). Analyzing of survey responses, we found that the most patients reported improved IBD symptoms (56%), gained understanding of their condition (84%) and were in favor of continuing STABILITY consultations during infusion therapy (93%). To further investigate the impact of STABILITY, we conducted a comparative analysis between IBD patients undergoing STABILITY infusion therapy and LSUHS patients solely on self-injectable biologics. Our paired data analysis showed significant improvements in disease severity in female IBD patients (1.69 ± 0.13 vs. 1.41 ± 0.12, *p* = 0.0001) and male IBD patients (1.58 ± 0.16 vs. 1.2 ± 0.135, *p* = 0.004), in UC patients (1.833 ± 0.4.2 vs. 1.444, *p* = 0.043), in all CD patients (1.59 ± 0.11 vs. 1.29 ± 0.01, *p* = 0.0001), in male CD patients (1.52 ± 0.167 vs. 1.15 ± 0.15, *p* = 0.016), in female CD patients (1.66 ± 0.15 vs. 1.4 ± 0.13, *p* = 0.0003), in female UC patients (1.82 ± 0.32 vs. 1.45 ± 0.31, *p* = 0.0379), and marginally in male UC patients (*p* = 0.0781). Similarly, hospitalizations were significantly reduced in CD patients considered in aggregate (0.21 ± 0.04 vs. 0.11 ± 0.03, *p* = 0.0013), in male IBD patients (0.175 ± 0.06 vs. 0.05 ± 0.035, *p* = 0.024), in female IBD patients (0.21 ± 0.05 vs. 0.11 ± 0.04, *p* = 0.0072), in male CD patients (0.18 ± 0.07 vs. 0.06 ± 0.042, *p* = 0.0436), and in females with CD (0.23 ± 0.06 vs. 0.13 ± 0.04, *p* = 0.013). Although average values for fecal calprotectin, CRP, and sedimentation rate were frequently reduced after STABILITY interviews, these data did not reach statistical significance. These preliminary findings suggest that STABILITY may be effective in maintaining low disease activity or remission in IBD patients.

## 1. Introduction

Inflammatory bowel disease (IBD), includes ulcerative colitis (UC) and Crohn’s disease (CD), chronic conditions characterized by gastrointestinal inflammation [1,2,3]. IBD has a worldwide prevalence of ~0.3% [4]. IBD management often involves biologic therapies, which have high clinical effect in controlling symptomatology and inducing remission [5,6,7]. However, the benefit of these therapies can vary among individuals, and determining those factors that contribute to treatment efficacy may optimize patient outcomes. It has been reported that enriched patient interactions have been correlated with improved patient satisfaction and clinical benefits [8,9,10,11]. In 2019, our Department of Gastroenterology initiated a care improvement approach for inflammatory bowel disease patients where a GI fellow was assigned to the infusion clinic each month to evaluate CD and UC patients when they arrived to receive their biologic medication infusions. Historically, these patients would see only the infusion nurse during their scheduled treatments; a GI physician was only present when requested by the patient or if they were visibly unwell and the nurse requested a physician. Our department recognized that many patients were not always on an acceptable disease trajectory. It was common for IBD patients to come for their infusions but would miss scheduled GI clinic visits and as a result, many patients who needed changes in their biologic therapy dosing or frequency (based on continued or worsening symptoms or high inflammation) went identified. We also found many IBD patients who were out of date for their colonoscopies or abdominal imaging for disease staging. We would even find that a few patients were presenting to our Emergency Department for flares related to their IBD without having any resultant changes made in their therapy plans. Subjectively, we felt that after we started to see these patients’ infusion clinic, our staff began making more frequent changes in medication and scheduling for several patients for procedures, and we also sensed that we were seeing fewer of these patients admitted to the hospital.

The purpose of this study was to investigate whether this approach for our infusion clinic resulted in significantly improved outcomes and if the frequency of hospital admissions was reduced, as well as to evaluate objective findings of disease severity, such as inflammatory markers and endoscopic mucosal healing. Our comprehensive analysis of patients’ demographics, levels of pertinent biomarkers (fecal calprotectin, c-reactive protein, and erythrocyte sedimentation rates), instances of hospitalization, medication changes, and dates of diagnosis—both prior and after the introduction of therapeutic regimen known as STABILITY—showed several improvements in disease severity and hospitalization outcomes, many of which were significant [11,12,13]. C-reactive protein (CRP) and erythrocyte sedimentation rate are standard clinical tests used to assess the severity of inflammation in IBD [14,15,16,17,18].

Sex differences in patients with inflammatory bowel disease (IBD) affect how the disease appears and how it is managed [19,20]. Consequently, we investigated whether and to what degree a systematic review of symptoms in patients receiving biologic therapy for IBD, referred to as ‘STABILITY’, influenced levels of fecal calprotectin (FCP) [12,13], C-reactive protein (CRP), and erythrocyte sedimentation rate as standard clinical tests used to assess the severity of inflammation in IBD [14,15,16,17,18].

## 2. Materials and Methods

This study included the population of IBD patients at OLSU who have ICD 10 coding for ulcerative colitis (UC) or Crohn’s disease (CD) and are currently receiving treatment at our infusion clinic. We excluded patients younger than 18 years old and prisoners. Our sample size was 111 patients. Our data comparing IBD disease states prior to and after the initiation of the fellow-run infusion clinic were collected from January 2017 to August 2020. The patients in our study were treated with infliximab, vedolizumab, certolizumab, or inflectra (biosimilar to infliximab). IBD patients who were treated with self-injected biologics (adalimumab, ustekinumab) were excluded since these self-injected they were not assessed by a clinician at the time of injection. The only injectable biologic included in this study was certolizumab, as we have several patients who present to infusion clinic to have the nurse administer this injection. We reviewed the electronic medical records of each patient, collecting data about their disease severity, number of hospitalizations/emergency room visits, and overall management. We also conducted a survey for each patient to assess their perception of their disease state and satisfaction with care. Our population of patients (111 total) was first divided into UC and CD groupings. We found we had 18 UC patients and 93 CD patients (16% and 84%). We collected all available objective markers of inflammation to assess disease severity in these patients. We included serum inflammatory markers (CRP and SED rate), stool calprotectin (a sensitive test for detecting inflammation in the stool), and any hospitalizations or emergency room visits before and after March 2019. Because we did not have endoscopic scores for all patients, we used a composite IBD score, which incorporated fecal calprotectin and CRP, and patients reported clinical improvements over a scale from 0 to 3, with 0 representing no disease activity and 3 representing severe disease. We assigned each patient a measure of disease severity, either mild, moderate, or severe, and collected the same data in these patients from March 2019 to August 2020 for comparison.

Statistics. We used paired Student’s *t*-testing to compare patients’ clinical findings before and after implementation of STABILITY when we had ‘before’ and ‘after’ findings for that patient and did not consider the groups as unpaired data sets. A *p*-value less than 0.05 was considered significant, and *p*-values between 0.05 and 0.1 were considered marginally significant. Standard error measures the precision of the sample mean as an estimate of the population mean. It indicates how much the sample mean is likely to vary from the true population mean. Means with standard error are reported in Table 1.

## 3. Results

We examined the impact of STABILITY on the reduction of disease severity, fecal calprotectin, C-reactive protein, sedimentation rate, and hospitalizations. Out of the CD patients, 36 (38.7%) had changes in their therapy or dosing; in UC, 6 patients (33%) underwent changes in therapeutic treatments or dosing. We considered these data for each form of IBD and considered how gender affects the outcomes for these conditions.

### 3.1. Ulcerative Colitis Patients

Demographics and Disease Severity: Our UC patients (18 total) were 23 - 64 years of age, (mean = 40 ± 3.2). 61% of UC cases were women with 39% men. Disease severity was 1.83 ± 0.26 before STABILITY, which significantly decreased after STABILITY (to 1.44 ± 0.22 (*p* = 0.043, paired two-tailed *t*-test). Clinical biomarkers associated with IBD disease severity were reduced but not significantly. The mean FCP level in UC prior to STABILITY was 1657.43 ± 724 µg/g; this decreased (not significantly) to 672 ± 314 µg/g after STABILITY (*p* = 0.5531). Mean CRP levels prior to STABILITY were 3.55 ± 1.3 mg/L; after STABILITY review, this decreased marginally significantly (*p* = 0.07) to 0.84 ± 0.17 mg/L. Sedimentation rates also decreased from 50.125 12.2 to 25.5 ± 5.4 (*p* = 0.20), which did not reach statistical significance. The mean number of hospitalizations in UC patients prior to STABILITY was 0.11 ± 0.07, and after STABILITY review, it was 0; the pfor change in hospitalizations was not statistically significant (*p* = 0.1631).

### 3.2. Crohn’s Disease Patients

Demographics and Disease Severity: Our CD patients (93 total) ranged from 16–67 years with 65% women, and 35% men. Disease severity was 1.6 ± 0.11 before STABILITY; this decreased significantly to 1.30 ± 0.097 after STABILITY (*p* = 0.0001). Mean FCP levels prior to STABILITY in CD patients were 464.11 ± 165 µg/g; after STABILITY review, it was reduced to 339 ± 112 µg/g (not significant, *p* = 0.2596). Mean CRP levels in CD prior to STABILITY were 2.05 ± 0.36 mg/L; after STABILITY, this was reduced to 1.49 ± 0.32 mg/L (not significant, *p* = 0.1957). STABILITY also reduced sedimentation rates from 26.7 ± 2.9 to 25.1 ± 4.6 albeit non-significantly (*p* = 0.86). Hospitalizations were reduced from 0.22 ± 0.04 prior to STABILITY to 0.10 ± 0.03, a significant reduction (*p* = 0.0013).

### 3.3. Female IBD Patients

When we evaluated women with IBD (a total of 71 patients) (mean age = 38.25 ± 1.8) we found several improvements in outcomes. Prior to STABILITY, disease severity was 1.69 ± 0.13 in female IBD patients, this was very significantly decreased by STABILITY (1.411 ± 0.12, *p* = 0.0001). IBD hospitalizations for women with IBD also significantly decreased after STABILITY (0.21 ± 0.05 to 0.11 ± 0.04, *p* = 0.0072). In women with IBD fecal calprotectin (FCP) decreased from 388 ± 137 μg/g (before STABILITY) to 268 ± 141 μg/g after STABILITY (not significant, *p* = 0.5522). Similarly, the average C-reactive protein (CRP) levels also went down following STABILITY (2.23 mg/L ± 0.42 to 1.37 ± 0.33 mg/L in the follow-up period (this was not significant, *p* = 0.14). In female IBD patients sedimentation rates also diminished albeit not significantly after STABILITY from 35.1 ± 4.1 to 30.2 ± 5.9 (not significant, *p* = 0.43). 

### 3.4. Male IBD Patients

Our study also evaluated male IBD patients as a group who had an average age of 41.1 ± 2.24 years. The mean disease severity for this group prior to STABILITY was 1.58 ± 0.16. This decreased very significantly to 1.2 ± 0.135 after STABILITY (paired *t*-test, *p* = 0.004). The mean FCP levels in these patients were lowered after STABILITY from 1079.0 ± 420 μg/g to 502.6 ± 163 μg/g; this was not significant (unpaired *p* = 0.4045). CRP levels were also reduced (from 2.6 ± 0.73 to 1.4 ± 0.45) again not significant, *p* = 0.14), and sedimentation rates (SED) were reduced from 21 ± 4 to 17.5 ± 5.1 after STABILITY (*p* = 0.3948). In male IBD patients, hospitalizations were significantly reduced from 0.175 ± 0.06 to 0.05 ± 0.03 (*p* = 0.0234).

### 3.5. Female CD Patients

We also evaluated women with CD (60 patients) who had an average age of 37.83 ± 1.9 years. In women with CD, mean disease severity prior to STABILITY was 1.66 ± 0.15, which decreased very significantly to 1.4 ± 0.13 following the STABILITY protocol (paired *t*-test, *p* = 0.0003). FCP levels decreased albeit not significantly from 339.25 ± 149 μg/g before STABILITY, to 277.4 ± 155 μg/g after STABILITY (*p* = 0.7693). Similarly, mean CRP was decreased but did not reach significance (from 2.25 mg/L to 1.56 mg/L (paired *p* = 0.3284). Sedimentation rates were reduced from 32.2 ± 3.7 to 30.7 ± 6.4 (*p* = 0.7) not statistically significant. However, IBD hospitalization rates for women with CD were significantly reduced from 0.23 ± 0.06 to 0.13 ± 0.5 following STABILITY (paired *t*-test, *p* = 0.013).

### 3.6. Male CD Patients

We also evaluated how STABILITY affected men with CD as a group (33 total). This group had an average age of 41.52 ± 2.6 years. Disease severity in men with CD was significantly decreased following STABILITY, from 1.50 ± 0.17 to 1.15 ± 0.15 (paired *t*-test, *p* = 0.016). In men with CD the average level of FCP was lower after STABILITY (before 699 ± 381 μg/g and after 426 ± 166 μg/g) but did not reach significance (paired *t*-test, *p* = 0.613). Mean CRP levels also decreased, again not significantly, paired *t*-test, *p* = 0.558) from 2.09 ± 0.8 mg/L to 1.4 ± 0.53 mg/L. Sedimentation rates also decreased after STABILITY, (not significant, paired *t*-test, *p* = 0.397) from 18.7 ± 4.5 to 16.9 ± 5.8. However, hospitalization for men with CD diagnoses was reduced significantly following STABILITY from 0.180 ± 0.07 to 0.06 ± 0.04 (paired *t*-test, *p* = 0.0435).

### 3.7. Female UC Patients

Our study also included women with ulcerative colitis (11 total patients) who had an average of 40.55 ± 4.7 years. In this group, mean disease severity prior to initiating STABILITY was 1.82 ± 0.32. After STABILITY, mean disease severity was significantly lower, 1.45 ± 0.31 (paired *t*-test, *p* = 0.0379). We also found that the mean FCP level was lowered from 778.33 ± 615.11 μg/g to 155 ± 139 μg/g after STABILITY but this was not a statistically significant change (*p* = 0.4931). Similarly, mean serum CRP levels also decreased from 2.08 ± 1.73 mg/L to 0.47 ± 0.05 mg/L following STABILITY; again not significant (*p* = 0.3995) and the sedimentation rate was also lowered from 80.7 ± 23.45 to 29.5 ± 5.5, not significant (*p* = 0.19) as were hospitalization rates which decreased from 0.91 ± 0.09 to 0 ± 0, but which were not significant (*p* = 0.34).

### 3.8. Male UC Patients

Our study included seven male UC patients with a mean age of 39.14 ± 3.9 years. We found a nearly significant change in disease severity from 1.86 ± 0.46 to 1.43 ± 0.3 (marginally significant, paired *t*-test, *p* = 0.0781). Mean FCP levels decreased from 2316.75 μg/g ± 1144 to 1189 ± 195 μg/g (not significant (*p* = 0.66). CRP levels in male UC patients were reduced by STABILITY from 4.6 ± 1.9 to 1.46 ± 0.3 (not significant, paired *t*-test value = 0.138). Average sedimentation rates in male UC were reduced by STABILITY from 31.8 ± 5.38 to 21.5 ± 21.5 ± 10.5, but statistical significance could not be determined in this group. Hospitalizations in male UC patients were also reduced by STABILITY, from 0.14 ± 0.14 to 0 ± 0 after STABILITY, but again, this did not reach statistical significance (*p* = 0.3559).

## 4. Discussions

The main observations we found in this study were consistent reductions in disease activity and hospitalizations, findings which were found in male and female IBD groups (including both CD and UC). Sex-based differences in IBD disease severity have been previously described [20]. Several scoring systems have been used to assess the activity of CD and UC. Commonly used indices in Crohn’s disease include the Crohn’s Disease Activity Index (CDAI), where scores range from 0 to 600, with higher scores indicating more severe disease activity. In UC, the Mayo Score is often employed, where scores range from 0 to 12, with higher scores indicating more severe disease activity [21]. Because we did not have endoscopic scores for all patients, we used a composite IBD score that incorporated fecal calprotectin and CRP and reported clinical improvements over a scale from 0 to 3, where 0 represents no disease activity and 3 represents severe disease. We found improvements in disease severity in UC (including males and females) and in female UC patients considered individually.

Improvements in hospitalization may be seen in CD as a result of the relatively larger number of individuals in these groups; smaller n-values in UC and missing values for paired analyses appear to have interfered with our ability to reach statistical significance, for example, disease severity in male UC approached (*p* = 0.0781) but did not reach statistical significance.

We acknowledge that normal values for plasma C-reactive protein (CRP), sedimentation rate, and fecal calprotectin [12,13,14,15] vary slightly among different institutions and testing methods; values are described below. Normal plasma CRP levels are typically less than 10 mg/L, and elevated CRP levels may indicate inflammation [12,15]. The normal sedimentation rate (ESR) [16,17,18] also varies by age and gender but, in general, typically falls below 20 mm/h for men and below 30 mm/h for women. Normal fecal calprotectin levels are usually <50 µg/g, and elevated FCP levels can suggest gastrointestinal inflammation in IBD. When clinical biomarkers for IBD were evaluated in our study, we saw reductions in the levels of FCP, CRP, and sedimentation rates; these did not reach statistical significance due to missing pairs and missing values, which were not always recorded in the medical records. In one case, in UC patients, CRP values were reduced marginally when considered as paired values (before and after STABILITY) (*p* = 0.0708); if all pre- and post-STABILITY values were considered (unpaired), we did reach the statistical significance of *p* = 0.0245 for this outcome.

### 4.1. Influence of Patient Sex on STABILITY Outcomes

Although sex differences in IBD patients have been shown to influence disease presentation and disease management in IBD [19,20], we did not observe a difference in response to STABILITY management of IBD. In our UC patients, our study included 18 patients, 61% of whom were female and 39% were male. Both male and female patients showed similar improvements in disease severity. In our study, CD patients had a sex distribution of 65% female and 35% male. There was a statistically significant decrease in both disease severity and hospitalizations after STABILITY in CD patients when males and females were considered together as well as when they were considered separately.

### 4.2. Patient Surveys

We recognize surveys as an important method to gain insights into patient IBD experiences and perceptions during biologic therapy [8]. Our survey explored several aspects related to treatment outcomes and the impacts of biologic therapy on patient lives. Our main findings from this survey were that patients thought they felt improvement during biologic therapy with STABILITY. In total, 72% of respondents reported experiencing a notable reduction in their IBD symptoms after initiating STABILITY, and 28% of patients expressed no significant change in symptoms or experienced exacerbation during the treatment period. An interesting result of our survey was that 85% of patients reported a better understanding of their condition after STABILITY, suggesting that STABILITY interactions with healthcare providers contributed to increased patient awareness and education about IBD. Similarly, 77% of our respondents described increased optimism about managing their condition and a better quality of life after STABILITY. Our survey also indicated that 62% of patients found the process of obtaining medication refills to be more straightforward and efficient compared to previous approaches. Our survey also showed that 48% of patients reported decreased ED visits since the initiation of STABILITY, indicating that this approach may contribute to better disease control and reducing acute exacerbations. This is supported by findings on reduced disease activity and reduced hospitalizations. Interactions with other IBD patients and IBD support groups can play a crucial role in coping with chronic conditions like IBD, and 36% of respondents expressed their intention to continue participating in support groups or seeking opportunities to connect with others who share similar experiences; 64% did not provide feedback on this topic.

## 5. Conclusions

In conclusion, this report shows how very brief reviews of symptoms in IBD patients being infused with biologics at an infusion center dramatically improved patient disease severity and hospitalizations. Our findings also suggest that lower, albeit not significant, levels of inflammatory biomarkers might help to explain these clinical reductions in disease activity and hospitalizations. Based on the costs of hospitalization for IBD (in 2014, hospitalization for CD cost USD 11,345 and USD 13,412 for UC) [22] and loss of income, STABILITY is a powerful and important platform that we feel should be applied where time constraints allow such interactions. Considering that in 2019, there were 23,000 hospitalizations for IBD [23], this would amount to a cost of USD 276 million dollars; we estimate this cost could be reduced by half, not even considering days of lost patient income and productivity. In programs where such patient interactions are not being applied, we anticipate that very brief meetings may provide enormously powerful adjustments that radically improve patient outcomes. Previous studies have shown that patients with chronic conditions treated with biologics experience better outcomes when they and their physicians are more actively engaged with their care [24,25,26].

We acknowledge several limitations to this preliminary study. First, we considered only how patients did before and after the implementation of this approach and did not accomplish an ‘untreated’ arm of patients who did not receive STABILITY interviews as the standard of care at our institution incorporated this approach for all infusion clinic patients receiving IBD care. Future studies should incorporate this group for comparisons. However, we believe that there were no other novel therapies that were added at the time of the ‘switch’ to STABILITY, which explains these benefits, but rather that the additional surveillance allowed physicians to make better decisions to guide therapy based on this approach.

Understanding the factors influencing therapy STABILITY and efficacy will contribute to improved patient care and treatment strategies in the future in both IBD patients and patients living with other chronic conditions such as lupus or rheumatoid arthritis.

## 6. Patents

Technology disclosures related to these findings and intellectual property reported in this manuscript have been submitted to the LSUHS Office of Sponsored Programs and Technology Transfer.

## Figures and Tables

**Table 1:** Tabular data from STABILITY.

Group		Parameter		Before	STABILITY		After	STABILITY		*p*-Value	Significance
Crohn’s	Disease	(CD)	Cohort		Patients	93					
	Age	Range	(years)	16	to	67					
	Female	(%)			N/A						
	Male	(%)			N/A						
	Mean	Disease	Severity	1.6	±	0.11	1.3	±	0.097	0.0001	Significant
	Mean	FCP	(μg/g)	464.11	±	165	339	±	112	0.2596	NS
	Mean	CRP	(mg/L)	2.05	±	0.36	1.49	±	0.32	0.1957	NS
	Sed Rate		(mm/h)	26.7	±	2.9	25.1	±	4.6	0.86	NS
	Hospitalizations			0.22	±	0.04	0.1	±	0.03	0.0013	Significant
Female	IBD	Patients			Patients	71					
	Mean	Age	(years)	38.25	±	1.8					
	Mean	Disease	Severity	1.69	±	0.13	1.411	±	0.12	0.0001	Significant
	Mean	FCP	(μg/g)	388	±	137	268	±	141	0.5522	NS
	Mean	CRP	(mg/L)	2.23	±	0.42	1.37	±	0.33	0.1642	NS
	Sed Rate		(mm/h)	35.1	±	4.1	30.2	±	5.9	0.43	NS
	Hospitalizations			0.21	±	0.05	0.11	±	0.04	0.0072	Significant
Male	IBD	Patients			Patients	40					
	Mean	Age	(years)	41.1	±	2.24					
	Mean	Disease	Severity	1.58	±	0.16	1.2	±	0.135	0.004	Significant
	Mean	FCP	(μg/g)	1079	±	420	502.6	±	163	0.4045	NS
	Mean	CRP	(mg/L)	2.6	±	0.73	1.4	±	0.45	0.14	NS
	Sed Rate		(mm/h)	21	±	4	17.5	±	5.1	0.3948	NS
	Hospitalizations			0.175	±	0.06	0.05	±	0.03	0.0234	Significant
Female	CD	Patients			Patients	60					
	Mean	Age	(years)	37.83	±	1.9					
	Mean	Disease	Severity	1.66	±	0.15	1.4	±	0.13	0.0003	Significant
	Mean	FCP	(μg/g)	339.25	±	149	277.4	±	155	0.7693	NS
	Mean	CRP	(mg/L)	2.25		1.56	0.3284				NS
	Sed Rate		(mm/h)	32.2	±	3.7	30.7	±	6.4	0.72	NS
	Hospitalizations			0.23	±	0.06	0.13	±	0.5	0.013	Significant
Male	CD	Patients			Patients	33					
	Mean	Age	(years)	41.52	±	2.6					
	Mean	Disease	Severity	1.5	±	0.17	1.15	±	0.15	0.016	Significant
	Mean	FCP	(μg/g)	699	±	381	426	±	166	0.613	NS
	Mean	CRP	(mg/L)	2.09	±	0.8	1.4	±	0.53	0.558	NS
	Sed Rate		(mm/h)	18.7	±	4.5	16.9	±	5.8	0.397	NS
	Hospitalizations			0.18	±	0.07	0.06	±	0.04	0.0435	Significant
Female	UC	Patients		of	Patients	11					
	Mean	Age	(years)	40.55	±	4.7					
	Mean	Disease	Severity	1.82	±	0.32	1.45	±	0.31	0.0379	Significant
	Mean	FCP	(μg/g)	778.33	±	615.11	155	±	139	0.4931	NS
	Mean	CRP	(mg/L)	2.08	±	1.73	0.47	±	0.05	0.3995	NS
	Sed Rate		(mm/h)	80.7	±	23.45	29.5	±	5.5	0.19	NS
	Hospitalizations			0.91	±	0.09	0	±	0	0.34	NS
Male	UC	Patients			Patients	7					
	Mean	Age	(years)	39.14	±	3.9					
	Mean	Disease	Severity	1.86	±	0.46	1.43	±	0.3	0.0781	Marginally
	Mean	FCP	(μg/g)	2316.75	±	1144	1189	±	195	0.66	NS
	Mean	CRP	(mg/L)	4.6	±	1.9	1.46	±	0.3	0.138	NS
	Sed Rate		(mm/h)	31.8	±	5.38	21.5	±	10.5		NS
	Hospitalizations			0.14	±	0.14	0	±	0	0.3559	NS

## Data Availability

For access to these data, please contact the corresponding author. Please note that all data requests are subject to privacy and ethical considerations review.
